# Predictors of functioning at clinical high-risk for psychosis

**DOI:** 10.1192/j.eurpsy.2022.304

**Published:** 2022-09-01

**Authors:** M. Omelchenko

**Affiliations:** Mental Health Research Centre, Youth Psychiatry, Moscow, Russian Federation

**Keywords:** Clinical high-risk, predictors of functioning level, Attenuated positive symptoms, Attenuated negative symptoms

## Abstract

**Introduction:**

In addition to the psychosis onset, patients at clinical high-risk (CHR) show a decrease of functioning. This may not be related to the degree and persistence of the attenuated positive symptom (APS). Other clinical factors also predict the level of remission.

**Objectives:**

Revealing the predictors of the functioning in the 5-year follow-up in patients at CHR.

**Methods:**

124 young depressive patients at CHR were examined. Depression symptoms were assessed on the HDRS scale, and the CHR symptoms were assessed on the SOPS scale. The follow-up examination was conducted after 5 years with the determination of functioning on the PSP scale. A correlative analysis of the predictors of the level of remission was conducted.

**Results:**

The functioning level was inversely related to the length of a depressive episode with the CHR symptoms (r=-0,432, p˂0.05), to the negative sub-scale SOPS score (r=0.312, p˂0.05) and to the symptoms of disorganization sub-scale SOPS score (r=0.246, p˂0.05) in the primary assessment. Insufficient reduction of the positive, negative symptoms and symptoms of disorganization on the SOPS during in-patient treatment was also a predictor of the worst outcome at the 5-year follow-up (r=-0,206, p˂0,05; r=-0,309, p˂0,05; r=-0,355, p˂0,05, and r=-0,349, p˂0,05, respectively).

**Conclusions:**

There are some factors, except the severity of APS, that may be considered as the predictors of functioning level in patients at CHR.

**Disclosure:**

No significant relationships.

